# Paneth Cell Ablation Aggravates Pancreatic and Intestinal Injuries in a Rat Model of Acute Necrotizing Pancreatitis after Normal and High-Fat Diet

**DOI:** 10.1155/2019/8474523

**Published:** 2019-11-11

**Authors:** Yuecheng Guo, Chunlan Huang, Liyan Liu, Xinyuan Fu, Yingying Lu, Junyuan Zheng, Qixiang Mei, Zehua Huang, Junjie Fan, Lungen Lu, Yue Zeng

**Affiliations:** ^1^Department of Gastroenterology, Shanghai General Hospital, Shanghai Jiao Tong University School of Medicine, Shanghai, China; ^2^Shanghai Key Laboratory of Pancreatic Diseases, Shanghai Jiao Tong University School of Medicine, Shanghai, China; ^3^International Medical Care Center, Shanghai General Hospital, Shanghai Jiao Tong University School of Medicine, Shanghai, China; ^4^Shanghai Minhang High School, Shanghai, China

## Abstract

We previously reported that acute necrotizing pancreatitis (ANP) after normal or high-fat diet is associated with a decreased number of Paneth cells in ileal crypts. Here, we ablated Paneth cells in a rat model of ANP after normal and high-fat diet to investigate the effects on disease symptoms. Adult male Sprague-Dawley rats received standard rat chow or a high-fat diet for 2 weeks, after which they were treated with dithizone to deplete Paneth cells. Six hours later, ANP was established by retrograde injection of sodium taurocholate into the biliopancreatic duct. Rats were sacrificed at 6, 12, and 24 h for assessment. We found dithizone aggravated ANP-associated pathological injuries to the pancreas and ileum in rats on high-fat or standard diets. Lysozyme expression in ileal crypts was decreased, while serum inflammatory cytokines (TNF*α*, IL-1*β*, and IL-17A) and intestinal permeability (serum DAO activity and D-lactate) were increased. Expression of tight junction proteins (claudin-1, zo-1, and occludin) was decreased. Using high-throughput 16S rRNA sequencing, we found dithizone reduced microbiota diversity and altered microbiota composition in rats on high-fat or standard diets. Dithizone decreased fecal short-chain fatty acids (SCFAs) in rats on high-fat or standard diets. Changes in intestinal microbiota correlated significantly with SCFAs, lysozyme, DAO activity, D-lactate, inflammatory cytokines, and pathological injury to the pancreas and ileum in rats on high-fat or standard diets. In conclusion, ablation of Paneth cells exacerbates pancreatic and intestinal injuries in ANP after normal and high-fat diet. These symptoms may be related to changes in the intestinal microbiota.

## 1. Introduction

Acute necrotizing pancreatitis (ANP) is a severe form of acute pancreatitis characterized by inflammation associated with necrosis in and around the pancreas. High-fat diet increases the risk of ANP [1] and can exacerbate ANP inflammation and increase complication rates [2]. Dysbiosis of intestinal microbiota has also been reported to correlate with a proinflammatory response in ANP patients [3]. We have previously reported that ANP associated with high-fat diet intake is associated with severe systemic inflammation, decreased Paneth cells in ileal crypts, altered microbiota composition, and decreased microbial diversity [4, 5]. However, the biological significance of these symptoms and their contribution to ANP are unclear.

Paneth cells are zinc-containing cells at the base of the intestinal crypts that play a vital role in intestinal barrier function [6]. Paneth cells regulate intestinal homeostasis by synthesizing and releasing antimicrobial peptides such as lysozyme and *α*-defensins [7], and thus, disrupting Paneth cell secretion can lead to inflammatory disease [8]. Specific deficiency of defensins excreted from Paneth cells during ileal Crohn's disease may compromise innate immune defenses of the ileal mucosa and perpetuate this disease [9]. A high-fat diet increases serum lipid levels [10] and decreases the number of Paneth cells, which in turn reduces the level of antimicrobial peptides in the intestine and alters microbiota structure [11]. Short-chain fatty acids (SCFAs) are produced by microbial fermentation of undigested dietary carbohydrates in the intestine [12] and can protect the intestinal barrier by upregulating epithelial tight junction proteins (TJPs) [13, 14].

In this study, we used dithizone, a chelator of zinc complexes [15], to deplete Paneth cells in rats with ANP after normal and high-fat diet. Effects on intestinal barrier function, microbiota structure, SCFAs, and pancreatic and ileal pathological injuries were examined.

## 2. Materials and Methods

### 2.1. Animals

Male Sprague-Dawley rats (4 weeks old) were obtained from Shanghai SLAC Laboratory Animal Co. (Shanghai, China). Rats were housed in groups of 5 at 24°C with a 12/12-hour light-dark cycle and given free access to water and chow. This study was carried out in accordance with the recommendations of the National Animal Protection Guidelines approved by the local animal ethics committee. The protocol was approved by the Animal Care and Use Committee of Shanghai Jiao Tong University.

### 2.2. Dithizone Treatment and ANP Induction

Rats in the standard diet groups were maintained on standard (STD) diet (Shanghai SLAC Laboratory Animal Co., China), while those in the high-fat- (HF-) fed groups were maintained on high-fat diet (STD diet supplemented with 20% lard and 3% cholesterol; Shanghai SLAC Laboratory Animal Co. China) [5, 16].

After 2 weeks of feeding, rats in each dietary group were randomly divided into 2 equal groups (*n* = 24 per group). Rats in the dithizone groups (DI+STD and DI+HF) were intravenously injected with 100 mg/kg dithizone (Sigma-Aldrich, USA), while nondithizone groups (STD and HF) were injected with an equal volume of saline. Six hours after injection, all rats were anesthetized by intraperitoneal injection of 0.05 mg/kg sodium pentobarbital (Shanghai Yuyan Instruments, China). Rats in each group were then infused with 3.5% sodium taurocholate solution (Sigma-Aldrich, USA) at a volume of 0.1 ml/100 g *via* the biliopancreatic duct at the speed of 0.2 ml/min to induce ANP [4]. Each group also included control rats that were given a sham biliopancreatic infusion of saline without ANP induction.

Rats were sacrificed by decapitation at 6, 12, and 24 h after infusion for histological assessment of the pancreas and distal ileum (*n* = 8 per treatment per time point). Blood samples were collected from the abdominal aorta. Segments of the distal ileum and the pancreas were isolated, flash-frozen in liquid nitrogen, and stored at -80°C. Freshly excreted feces were also collected from rats for analysis of SCFAs before they were anesthetized.

### 2.3. Histological Analysis

Pancreatic and distal ileal tissues were fixed in 4% paraformaldehyde, dehydrated, embedded in paraffin, and cut into 4 *μ*m sections. Sections were stained with hematoxylin and eosin (H&E, Servicebio, China) and examined under a light microscope (DM5500 B, LEICA, Germany) by two pathologists who were blinded to treatment group. Pancreatic injury was scored using criteria reported by Schmidt et al. [17], while injury to the distal ileum was scored as described by Chiu et al. [18]. Paneth cells were counted in at least 10 ileal crypts per section. Three histological sections were analyzed per animal in the histological analyses.

### 2.4. Serum Biochemistry

Serum triglyceride (TG) and total cholesterol (TC) levels were analyzed using the TG and T-CHO kits (product nos. A110-1 and A111-1, Nanjing Jiancheng Bioengineering Institute, Nanjing, China) according to manufacturer's protocols. D-lactate concentration was determined using the D-LA ELISA kit (Nanjing Jiancheng Bioengineering Institute). DAO activity was measured using the diamine oxidase (DAO) assay kit (Nanjing Jiancheng Bioengineering Institute) according to previously published protocols [5].

### 2.5. ELISA

Serum TNF*α*, IL-1*β*, and IL-17 levels were measured using the TNF Alpha Rat ELISA kit, IL-1*β* Rat ELISA kit, and IL-17A Rat ELISA kit (eBioscience, USA) according to manufacturer's protocols. All samples were measured in duplicate.

### 2.6. Immunohistochemistry

Tissue sections from the distal ileum were deparaffinized, and the antigens were retrieved with EDTA antigen retrieval buffer (pH 9.0). After extensive washing in phosphate-buffered saline (PBS, pH 7.4), the sections were quenched with 3% hydrogen peroxide, blocked with 3% bovine serum albumin at room temperature for 30 min, and incubated with 1 : 100 dilutions of primary antibody against claudin-1 (Abcam, USA), ZO-1 (Proteintech Group, USA), or occludin (Abcam, USA) at 4°C overnight. The next day, the sections were washed and incubated with horseradish peroxidase-labeled secondary antibody (1 : 200, Servicebio, Wuhan, China) for 50 min at room temperature. The slides were placed in PBS, washed 3 times on a decolorizing shaker for 5 min per wash, and visualized with diaminobenzidine (1 : 100, 50 ul per slide; DAB, DAKO, Denmark). Slides were visualized using a light microscope (DM5500 B, LEICA, Germany), and images were analyzed using Image-Pro Plus 6.0 (Media Cybernetics, USA). Three histological sections were analyzed per animal in the immunohistochemical analyses.

### 2.7. Western Blot

Tissue samples from the distal ileum were homogenized in RIPA lysis buffer containing 1% protease inhibitor (Beyotime, China), centrifuged at 10000 g for 10 minutes at 4°C and heated at 100°C for 10 minutes. Protein (30 *μ*g) was then loaded into a 10% SDS-PAGE gel for electrophoretic separation and transferred to a PVDF membrane. The membrane was blocked with 5% fat-free milk for 2 h and incubated with 1 : 1000 dilutions of antibodies against *β*-actin (Proteintech Group), claudin-1 (Abcam, USA), ZO-1 (Proteintech Group, USA), and occludin (Abcam, USA) overnight at 4°C. The next day, the membrane was washed 4 times with Tris-buffered saline with Tween-20 (TBST) buffer and incubated with peroxidase-conjugated AffiniPure Goat Anti-Rabbit secondary antibody (Jackson ImmunoResearch, USA) for 90 min at room temperature. Protein bands were visualized with chemiluminescent HRP Substrate (Immobilon, USA). Protein concentration was measured using a BCA kit (YEASEN, China).

### 2.8. RT-PCR

Total RNA was isolated from the distal ileum using TRIzol^TM^ (Invitrogen, USA). Reverse transcription was performed using the PrimeScript RT master mix kit (TaKaRa, Japan) and RT-PCR was performed using Premix Taq^TM^ (TaKaRa, Japan). Gene expression was quantified using the 2-*ΔΔ*Ct method. Primer sequences against the lysozyme gene were as follows: forward 5′-AGGAATGGGATGTCTGGCTAC-3′ and reverse 5′-GGTATCCCACAGGCGTT CTT-3′ (Sangon, China) [5]. *β*-Actin was used as an internal control. All experiments were performed in triplicate.

### 2.9. Immunofluorescence

Tissue sections were deparaffinized and rehydrated. Antigen retrieval was performed in citrate buffer, and endogenous peroxidase activity was blocked using 3% hydrogen peroxide in methanol for 20 min. Sections were incubated with a primary antibody against lysozyme (1 : 1000 dilution; DAKO) [4] at 4°C overnight. After washing, the sections were incubated with fluorescein-labeled secondary antibody (1 : 400 dilution; Servicebio, Wuhan, China) for 30 min. Sections were stained with DAPI for 5 min to visualize nuclei.

### 2.10. 16s rRNA Sequencing and Analysis

Microbial genomic DNA was extracted from 500 mg of feces from each rat using the EZNA® Soil DNA Kit (Omega BioTek, USA). DNA concentration was determined using a NanoDrop2000 (Thermo Scientific, USA). DNA integrity was verified by electrophoresis on a 1% agarose gel. The V3-V4 region of the bacterial 16S rRNA gene was amplified using the universal bacterial primers 338F (ACTCCTACGGGAGGCAGCA) and 806R (GGACTACHVGGGTWTCTAAT) (Sangon Biotech, China) [19]. PCR reactions were performed in a total volume of 20 *μ*l containing 10 ng DNA template, 4 *μ*l FastPfu buffer, 2 *μ*l dNTPs (2.5 mM), 0.8 *μ*l each primer (5 *μ*M), and 0.4 *μ*l of FastPfu polymerase (TransGen BioTech, Beijing, China). PCR cycles were as follows: 95°C for 3 min, followed by 25 cycles at 95°C for 30 s, 55°C for 30 s, and 72°C for 30 s, and a final extension at 72°C for 5 min. PCR products were analyzed by electrophoresis in a 2% agarose gel.

Equimolar concentrations of amplicons were sequenced on the Illumina MiSeq platform (Illumina, USA). High-quality sequences were assigned to the samples according to barcodes. A total of 3,450,879 high-quality sequences and 1627 operational taxonomic units (OTUs) were obtained from 64 samples. OTUs were clustered at 97% nucleotide similarity level using UPARSE (version 7.1, http://drive5.com/uprase/) [20], and chimeric sequences were identified using UCHIME (http://www.drive5.com/uchime/uchime_download.html) [21]. The taxonomy of each 16S rRNA gene sequence was analyzed using RDP Classifier2 (version 2.2, http://sourceforfe.net/projects/rdp-classifier/) against the SILVA 128 16S rRNA database (https://www.arb-silva.de/) [22, 23]. OTUs were used to calculate *α*-diversity estimations, including diversity (Shannon, Simpson), richness (Ace, Chao-1, Sobs), and Good's coverage and rarefaction curve analysis using Mothur (version 1.30.2, http://www.mothur.org/wiki/Schloss_SOP#Alpha_diversity) [20]. Principal component analysis (PCA) was performed on OTUs by the Euclidean method, and a heatmap of gene expression was generated using the R gplot package (version 3.3.1). Analysis of linear discriminant analysis effect size (LEfSe) was performed to explore distinctive bacteria at various levels using a linear discriminant analysis (LDA) threshold of >3.0; the cladogram is displayed according to effective size. Comparative analysis between groups was performed using a nonparametric Wilcoxon test. A correlation heatmap was used to investigate correlations among SCFAs, intestinal barrier dysfunction, pathological injuries, TC, TG, lysozyme levels, and gut microflora. Phylogenetic investigation of communities by reconstruction of unobserved states (PICRUSt) was used to predict microbial functional features based on 16S rRNA high-throughput sequencing data [24].

### 2.11. Analysis of Fecal SCFAs

Approximately 10 mg of feces was mixed with 500 *μ*l methanol and 10 *μ*l internal standard (2-ethylbutyric acid) (Darmstadt, Germany). The mixture was ground up in a tissue grinder at 65 Hz for 120 s, vortexed for 30 s, and then centrifuged at 10000 g for 15 min at 4°C. Supernatant (400 *μ*l) was then transferred to a 1.5 ml tube, mixed with 50 *μ*l methanol, and centrifuged at 10000 g for 15 min at 4°C. The supernatant was aspirated and analyzed by gas chromatography-mass spectrometry using an Agilent 6890A/5973C GC-MS platform (Agilent Technologies, USA) with a mass spectrometric range of 30-150 (m/z) in a full-scan mode. Feature extraction and preprocessing were done using the XCMS package in R (version 2.13.2) [25], and then the data were normalized and edited into a two-dimensional data matrix in Microsoft Excel 2010.

### 2.12. Statistical Analysis

Data are presented as mean ± standard deviation (SD). The Shapiro-Wilk test was used to test normality, and Levene's test was used to test homogeneity of variance. Comparisons between two groups were performed using the unpaired *T*-test (for normally distributed data) or Wilcoxon test (for skewed data). Spearman's rank correlation coefficient was used to test for correlations between the 50 most abundant bacterial species and biomarkers. For three or more groups, one-way ANOVA was preformed followed by Bonferroni's posttest for data with *F* at *p* < 0.05. All analyses were performed in SPSS 20.0 (IBM, Chicago, IL, USA). A *p* value of <0.05 was considered statistically significant.

## 3. Results

### 3.1. Dithizone Depletes Paneth Cells and Lysozyme in the Ileum

**Figure 1:**
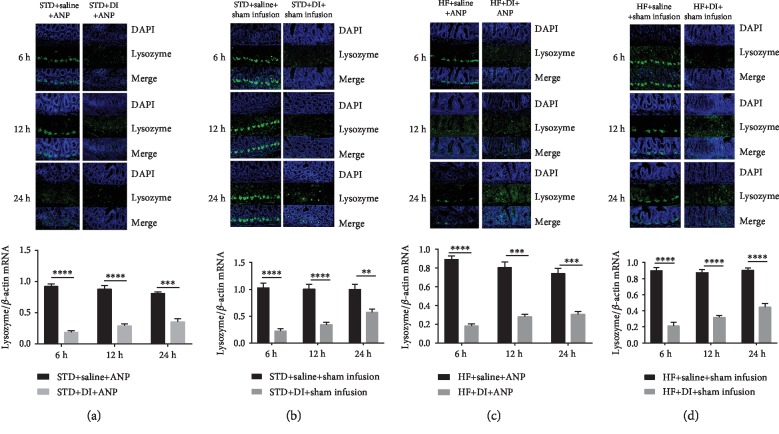
Dithizone depletes lysozyme in ileal crypts in rats. Lysozyme protein expression (green) in Paneth cells of the distal ileum as assessed by immunofluorescence. Nuclei were counterstained with DAPI (magnification ×200). Lysozyme mRNA expression as assessed by RT-PCR. (a) STD+saline+ANP vs. STD+DI+ANP. (b) STD+saline+sham infusion vs. STD+DI+sham infusion. (c) HF+saline+ANP vs. HF+DI+ANP. (d) HF+saline+sham infusion vs. HF+DI+sham infusion. ^∗^*p* < 0.05, ^∗∗^*p* < 0.01, ^∗∗∗^*p* < 0.001, ^∗∗∗∗^*p* < 0.0001, Student's *t*-test. ANP: acute necrotizing pancreatitis; DI: dithizone; HF: high-fat diet; STD: standard diet. Results were expressed as the mean ± SD (*n* = 8).

A pilot experiment showed that the number of Paneth cells was lowest at 6 h after dithizone treatment in rats on a standard or high-fat diet (Supplementary [Supplementary-material supplementary-material-1]). We therefore chose to perform retrograde sodium taurocholate infusion 6 h after dithizone treatment. At all time points, rats in all dietary groups treated with dithizone had lower lysozyme expression than those not treated with dithizone (Figure 1).

### 3.2. High-Fat Diet Exacerbates Injuries Caused by Retrograde Sodium Taurocholate Infusion

HF rats had higher levels of serum TG (0.627 ± 0.088 vs. 0.202 ± 0.048, *p* < 0.001) and TC (3.146 ± 0.711 vs. 1.414 ± 0.272, *p* < 0.001) than STD rats (Supplementary [Supplementary-material supplementary-material-1]). Serum TNF-*α*, IL-1*β*, and IL-17A levels and pancreatic injury scores were also higher in HF rats than STD rats at all time points assayed, suggesting exacerbation of ANP-associated pancreatic injury and systemic inflammation (Supplementary [Supplementary-material supplementary-material-1] and [Supplementary-material supplementary-material-1]).

**Figure 2:**
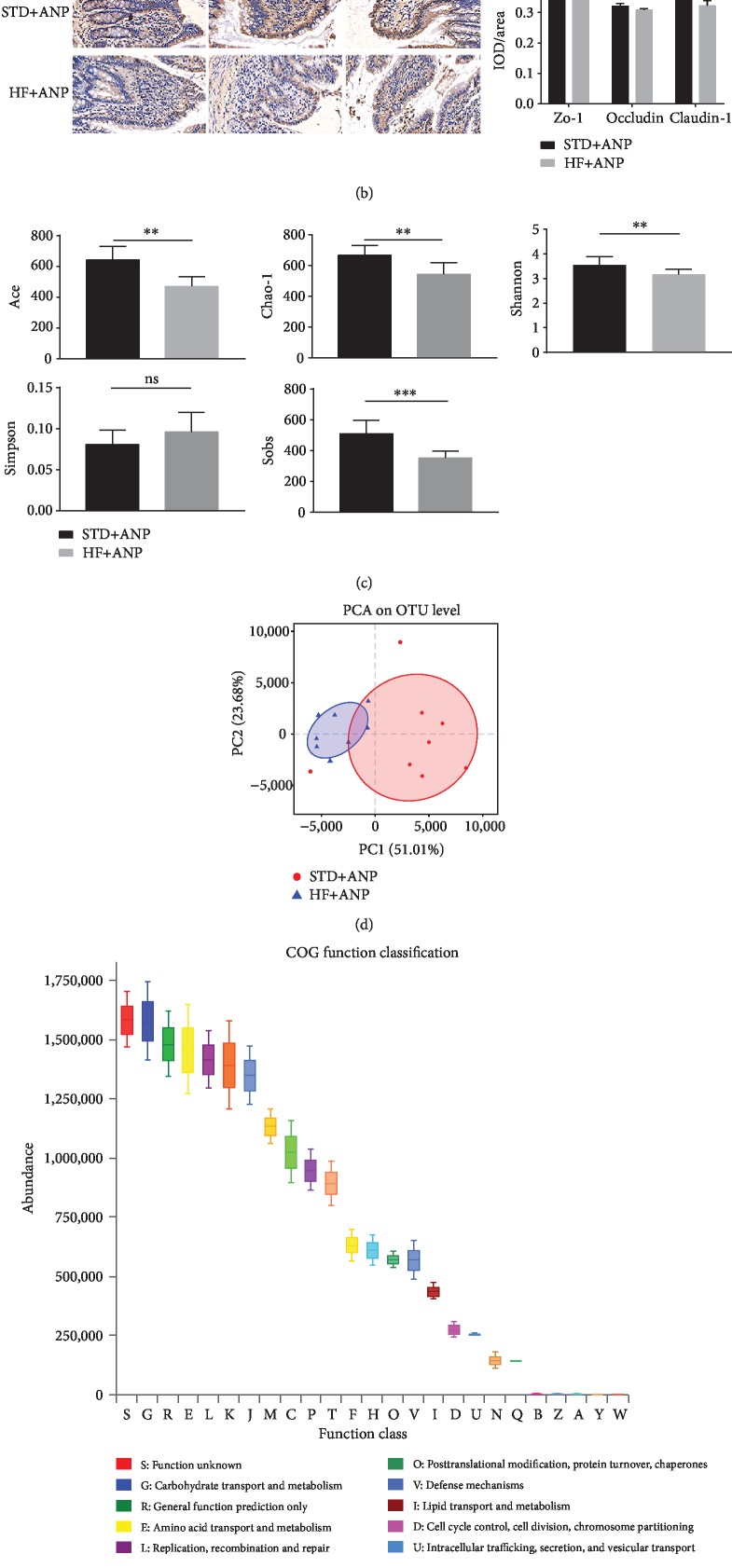
Effect of high-fat diet on ANP-associated injuries (TJPs and microbiota diversity) caused by retrograde sodium taurocholate infusion. Expression of caudin-1, occludin, and zo-1 as assessed by (a) western blot and (b) immunohistochemistry of the distal ileum. (c) Estimators of intestinal microbiota *α*-diversity in rats fed a high-fat or standard diet (*n* = 8). (d) Principal component analysis of microbiota *β*-diversity (*n* = 8). (e) Predicted microbial functional categories of 16S rRNA in fecal samples at 24 h after induction of ANP.^∗^*p* < 0.05, ^∗∗^*p* < 0.01, ^∗∗∗^*p* < 0.001, Student's *t*-test.

Compared to rats in the STD group, rats in the HF group had higher ileum injury scores (6 ± 0.707 vs. 3.3 ± 0.447, *p* < 0.001) (Supplementary [Supplementary-material supplementary-material-1] and [Supplementary-material supplementary-material-1]), increased DAO activity (1332.2102 ± 136.659 vs. 906.8835 ± 66.453, *p* < 0.001) and serum D-lactate (6625.0142 ± 501.387 vs. 5903.5772 ± 276.515, *p* = 0.022) (Supplementary [Supplementary-material supplementary-material-1]), and decreased expression of lysozyme (Supplementary [Supplementary-material supplementary-material-1] and [Supplementary-material supplementary-material-1]) and TJPs in the distal ileum (Figures 2(a) and 2(b) and Supplementary [Supplementary-material supplementary-material-1]) at 24 h.

Rats in the HF group had lower Sobs, Ace, Chao-1, and Shannon indexes than rats in the STD group, suggesting lower microbiota diversity (Figure 2(c)). PCA also revealed that high-fat feeding induced alterations in the structure of intestinal microbiota, as demonstrated by clustering and separation of HF and STD samples (Figure 2(d)).

**Figure 3:**
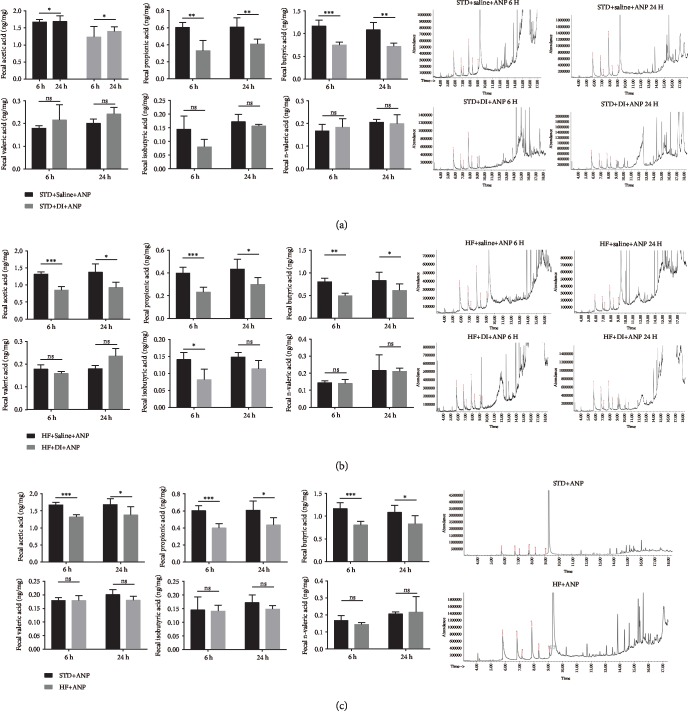
Fecal SCFAs levels as detected by gas chromatography-mass spectrometry. (a) Effects of dithizone on short-chain fatty acids in rats on standard diet followed by retrograde sodium taurocholate infusion. (b) Effects of dithizone on short-chain fatty acids in rats on high-fat diet followed by retrograde sodium taurocholate infusion. (c) Effects of high-fat diet on short-chain fatty acids in rats followed by retrograde sodium taurocholate infusion. ^∗^*p* < 0.05, ^∗∗^*p* < 0.01, ^∗∗∗^*p* < 0.001, Student's *t*-test. Results were expressed as the mean ± SD (*n* = 5).

The most enriched predicted functional category of 16s rRNA was carbohydrate transport and metabolism (Figure 2(e)). HF rats had lower levels of fecal SCFAs, including acetic acid, propionic acid, and butyric acid, than STD rats at 6 and 24 h (Figure 3(c)).

### 3.3. Dithizone Exacerbates ANP-Related Injury, Inflammation, and Intestinal Permeability in Rats on Standard Diet

**Figure 4:**
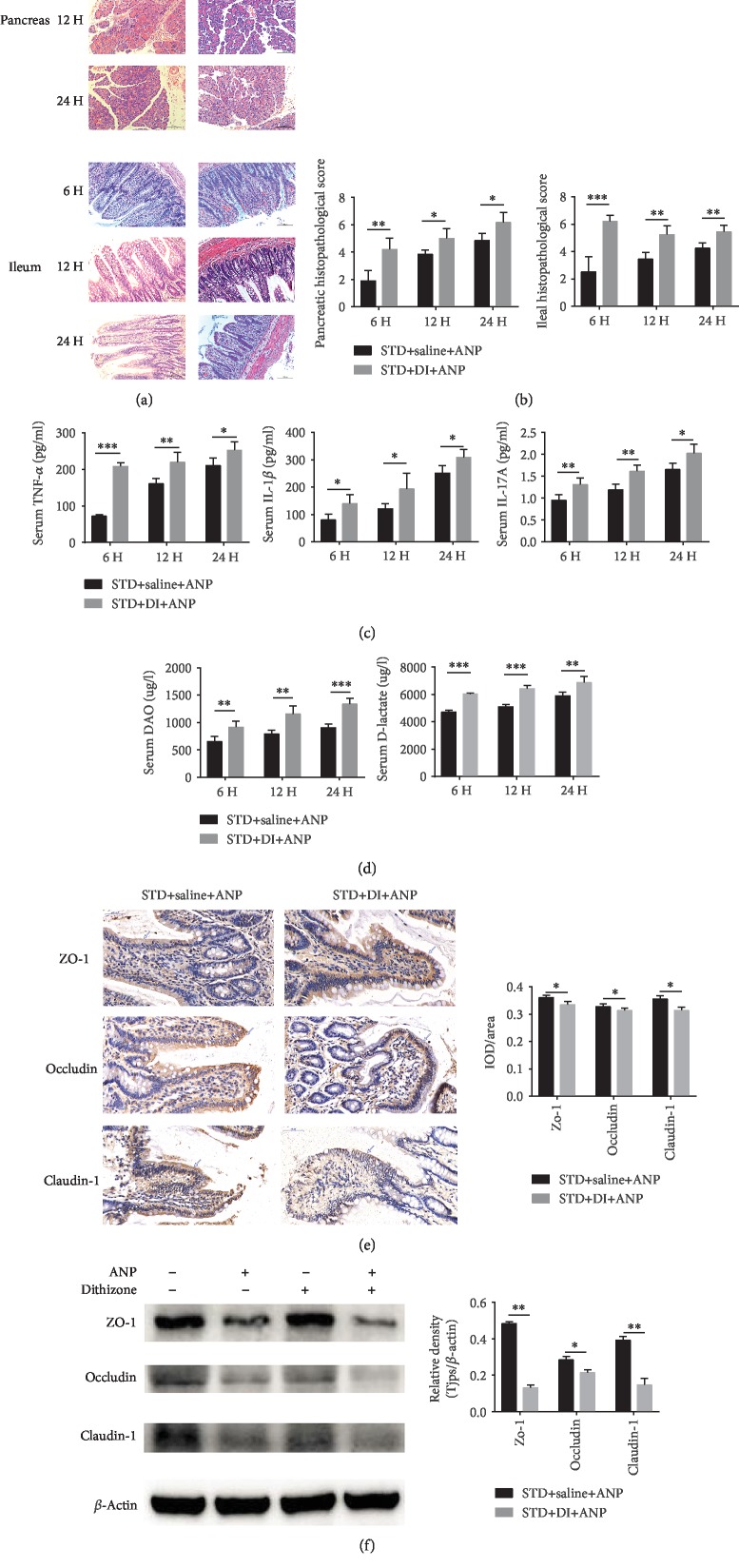
Effects of dithizone on pancreatic and intestinal injuries in rats on standard diet followed by retrograde sodium taurocholate infusion. (a) Pancreas and distal ileum sections of rats treated with dithizone or saline. Tissue was stained with H&E (magnification ×200). (b) Histopathological scores of the pancreas and distal ileum. (c) Serum TNF*α*, IL-1*β*, supplementary, and IL-17A levels in rats treated with dithizone or saline. (d) DAO activity and serum D-lactate in rats treated with dithizone or saline. (e) Distal ileum sections showing immunostaining for claudin-1, occludin, and zo-1, together with quantification of claudin-1, occludin, and zo-1 staining. (f) Expression of claudin-1, occludin, and zo-1 as assessed by western blot. ^∗^*p* < 0.05, ^∗∗^*p* < 0.01, ^∗∗∗^*p* < 0.001, Student's *t*-test. Results were expressed as the mean ± SD (*n* = 5).

Rats in the DI+STD group had exacerbated pancreatic and ileal injuries (Figures 4(a) and 4(b)) and higher expression of serum inflammatory cytokines (Figure 4(c)) than rats in the STD group at all time points. DAO activity and serum D-lactate levels (Figure 4(d)) were also higher in DI+STD rats than in STD rats, suggesting increased intestinal permeability. Expression of zo-1, occludin, and claudin-1 was decreased at 24 h (Figures 4(e) and 4(f) and Supplementary [Supplementary-material supplementary-material-1]). None of these parameters was significantly different between DI+STD and STD rats if ANP was not induced (Supplementary [Supplementary-material supplementary-material-1]).

### 3.4. Dithizone Reduces Microbial Diversity in Rats with ANP on Standard Diet

**Figure 5:**
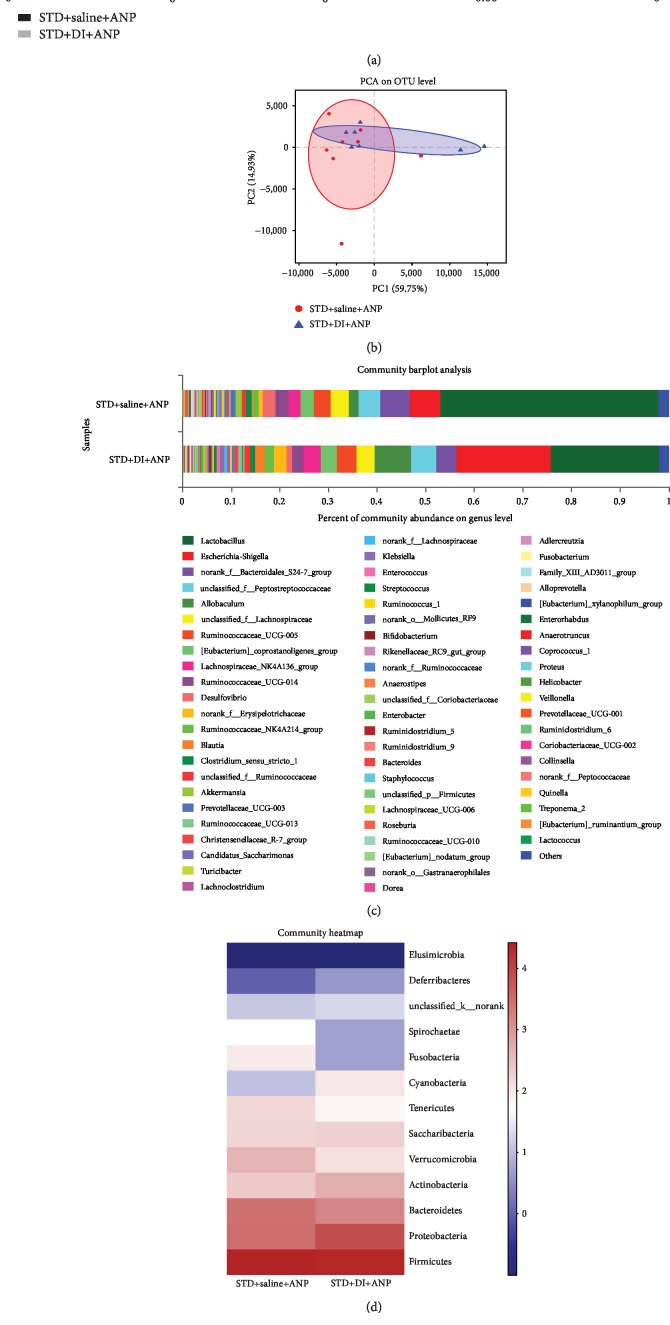
Effects of dithizone on diversity of fecal microbiota in rats on standard diet followed by retrograde sodium taurocholate infusion. (a) Estimators of intestinal microbiota *α*-diversity in rats treated with dithizone or saline. (b) Principal component analysis of intestinal microbiota *β*-diversity in rats treated with dithizone or saline. (c) Relative abundance of bacterial genera in rats treated with dithizone or saline. (d) Relative abundance heatmap of bacterial phyla in intestinal microbiota of rats treated with dithizone or saline. ^∗^*p* < 0.05, ^∗∗^*p* < 0.01, ^∗∗∗^*p* < 0.001, Student's *t*-test. *n* = 8 for each group.

Rats in the DI+STD group had lower Sobs, Ace, Chao-1, and Shannon indexes and a higher Simpson index than those in the STD group (Figure 5(a)), suggesting decreased microbiota diversity. PCA analysis showed that the microbiota composition in DI+STD and STD rats was distinct (Figure 5(b)). PC1 accounted for 59.75% of total variance, while PC2 accounted for 14.93% of total variance.

**Figure 6:**
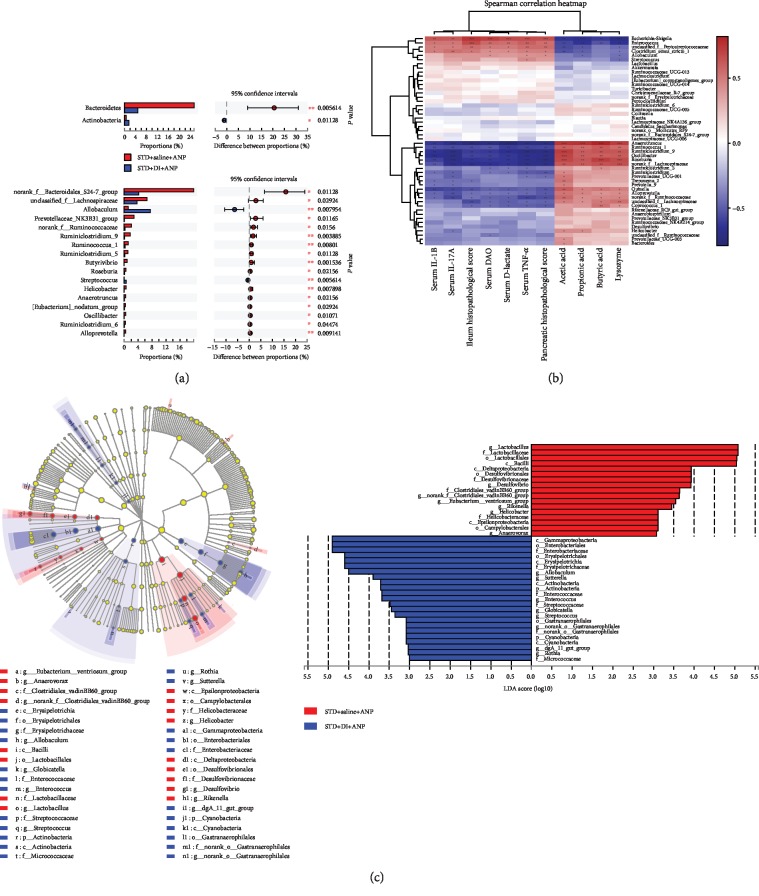
Effects of dithizone on microbiota composition in rats on standard diet followed by retrograde sodium taurocholate infusion. (a) Bacterial phyla and genera with significantly different abundance in rats treated with dithizone or saline. ^∗^*p* < 0.05, ^∗∗^*p* < 0.01, ^∗∗∗^*p* < 0.001, Wilcoxon test. (b) Heatmap showing correlation among SCFAs, lysozyme expression, intestinal barrier dysfunction, serum lipid, pathological changes, and gut microflora. Blue indicates a negative correlation, while red indicates a positive correlation. ^∗^*p* < 0.05, ^∗∗^*p* < 0.01, ^∗∗∗^*p* < 0.001, Spearman test. (c) Analysis of linear discriminant analysis effect size (LEfSe) using multilevel species hierarchy tree and LEfSe bars determined by linear discriminant analysis (LDA). *n* = 8 for each group.

The fecal microbial composition of DI+STD and STD rats is shown in Figures 5(c) and 5(d). Compared to rats in the STD group, rats in the DI+STD group showed enrichment of *Gammaproteobacteria* (from class to family), *Cyanobacteria* (from phylum to genus), *Sutterella* (genus), *Actinobacteria* (from phylum to class), *Rothia* (genus), *Erysipelotrichia* (from class to family), *Allobaculum* (genus), *Globicatella* (genus), *Streptococcus* (genus), and *Enterococcus* (genus) bacteria (Figure 6(c)). We also found significant enrichment of *Epsilonproteobacteria* (from class to genus), *Deltaproteobacteria* (from class to genus), *Anaerovorax* (genus), Bacilli (class), Rikenella (genus), and *Lactobacillaceae* (from family to genus) in STD rats compared to DI+STD rats. At the phylum level, DI+STD rats had fewer *Bacteroidetes* and more *Actinobacteria* than STD rats (Figure 6(a)). At the genus level, the following genera were lower in DI+STD rats than STD rats: *Prevotellaceae*_NK3B31_group, *Ruminiclostridium*_9, *Ruminococcus*_1, *Ruminiclostridium*_5, *Butyrivibrio*, *Roseburia*, *Helicobacter*, *Anaerotruncus*, *Oscillibacter*, *Ruminiclostridium*_6, and *Alloprevotella* (Figure 6(a)). DI+STD rats also had more *Allobaculum* and *Streptococcus* than STD rats (Figure 6(a)). Rats in the DI+STD group had lower fecal SCFA levels than those in the STD group at 6 and 24 h (Figure 3(a)).

When ANP was not induced (sham induction), DI+STD rats showed a trend towards lower intestinal microbiota diversity than STD rats, but this was not statistically significant (Supplementary [Supplementary-material supplementary-material-1]). PCA showed that DI+STD and STD rats still had distinct microbial compositions without induction of ANP (Supplementary [Supplementary-material supplementary-material-1]). However, there was no significant difference in fecal SCFA levels at 6 and 24 h (Supplementary [Supplementary-material supplementary-material-1]).

Spearman's rank correlation showed that *Alloprevotella*, *Quinella*, *Roseburia*, *Oscillibacter*, *Ruminococcus*_1, *Anaerotruncus*, and *Ruminiclostridium*_9 all positively correlated with fecal SCFAs and lysozyme expression, while they negatively correlated with serum DAO, serum D-lactate, serum inflammatory cytokines, and pathological injuries (Figure 6(b)). In contrast, *Escherichia-Shigella* and *Enterococcus* were negatively associated with SCFAs and lysozyme expression, while they positively associated with serum DAO, serum D-lactate, serum inflammatory cytokines, and pathological injuries (Figure 6(b)).

### 3.5. Dithizone Exacerbates ANP-Related Injuries, Inflammation, and Intestinal Permeability in Rats on High-Fat Diet

**Figure 7:**
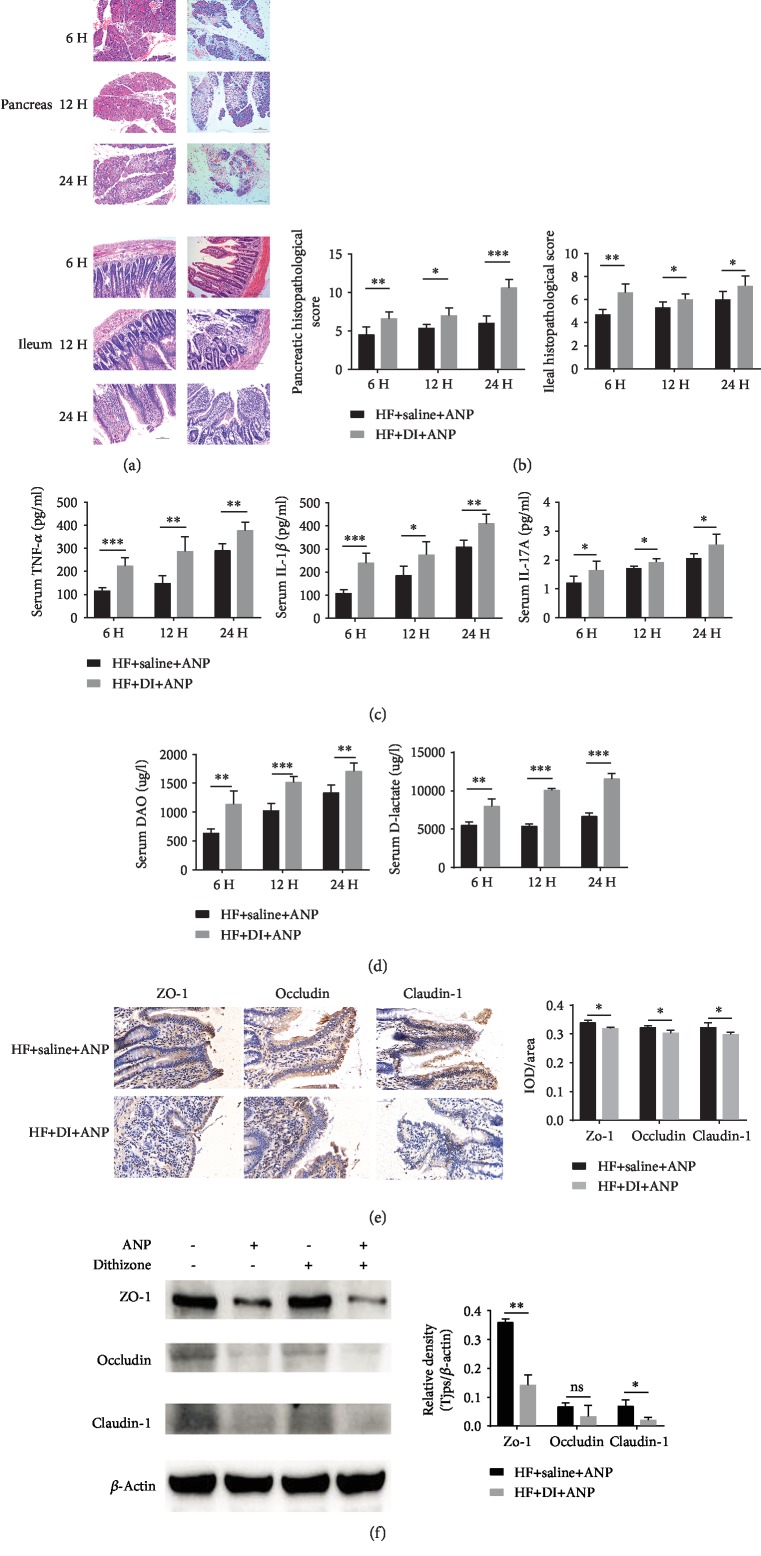
Effects of dithizone on pancreatic and intestinal injuries in rats on high-fat diet followed by retrograde sodium taurocholate infusion. (a) Sections of the pancreas and the distal ileum stained with H&E in rats treated with dithizone or saline (magnification ×200). (b) Histopathological scores of the pancreas and distal ileum. (c) Serum inflammatory cytokines in rats treated with dithizone or saline. (d) DAO activity and serum D-lactate in rats treated with dithizone or saline. (e) Sections of distal ileum showing immunostaining for claudin-1, occludin, and zo-1 in rats treated with dithizone or saline, together with quantification of claudin-1, occludin, and zo-1 immunostaining. (f) Expression of intestinal epithelial TJPs as assessed by western blot. ^∗^*p* < 0.05, ^∗∗^*p* < 0.01, ^∗∗∗^*p* < 0.001, Student's *t*-test. Results were expressed as the mean ± SD (*n* = 5).

Rats in the DI+HF group had higher pancreatic injury scores (Figures 7(a) and 7(b)) and higher serum levels of TNF-*α*, IL-1*β*, and IL-17A (Figure 7(c)) than rats in the HF group at all time points. DI+HF rats also had higher ileal injury scores (Figures 7(a) and 7(b)), higher DAO activity and serum D-lactate (Figure 7(d)), and decreased zo-1 and claudin-1 expression at 24 h (Figures 7(e) and 7(f) and Supplementary [Supplementary-material supplementary-material-1]). There was no difference in any of these parameters between DI+HF and HF rats if ANP was not induced (Supplementary [Supplementary-material supplementary-material-1]).

### 3.6. Dithizone Reduces Microbial Diversity in Rats with ANP on High-Fat Diet

**Figure 8:**
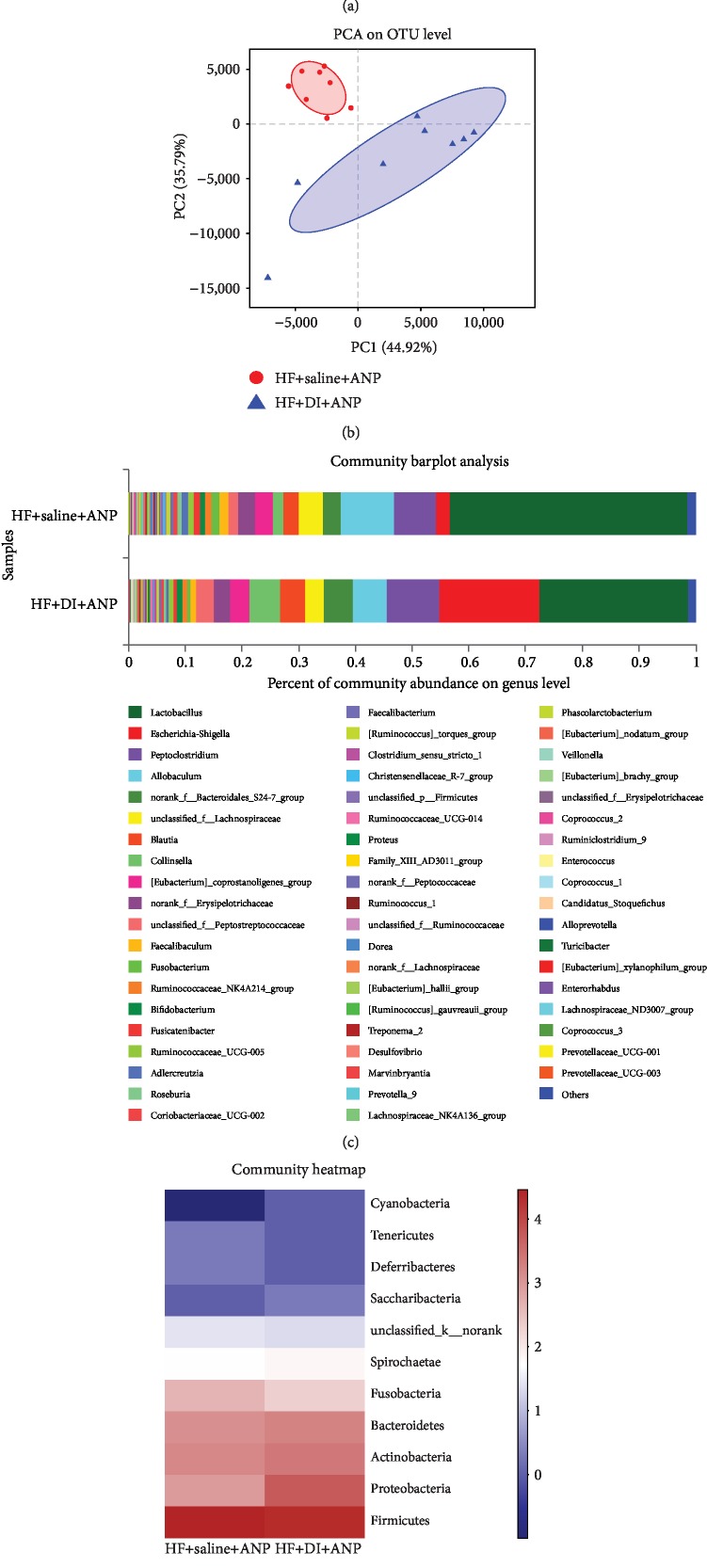
Effects of dithizone on microbiota diversity in rats on high-fat diet followed by retrograde sodium taurocholate infusion. (a) Estimators of intestinal microbiota *α*-diversity in rats treated with dithizone or saline. (b) PCA of microbial *β*-diversity in rats treated with dithizone or saline. (c) Relative abundance of intestinal microbial genera in rats treated with dithizone or saline. (d) Heatmap of relative abundance of bacterial phyla in intestinal microbiota of rats treated with dithizone or saline. ^∗^*p* < 0.05, ^∗∗^*p* < 0.01, ^∗∗∗^*p* < 0.001, Student's *t*-test. *n* = 8 for each group.

DI+HF rats had lower Sobs, Ace, Chao-1, and Shannon indexes and a higher Simpson index than HF rats, suggesting reduced microbiota diversity (Figure 8(a)). PCA analysis showed that the fecal microbial composition of DI+HF and HF rats was distinct (Figure 8(b)). PC1 accounted for 44.92% of total variance, and PC2 accounted for 35.79% of total variance.

**Figure 9:**
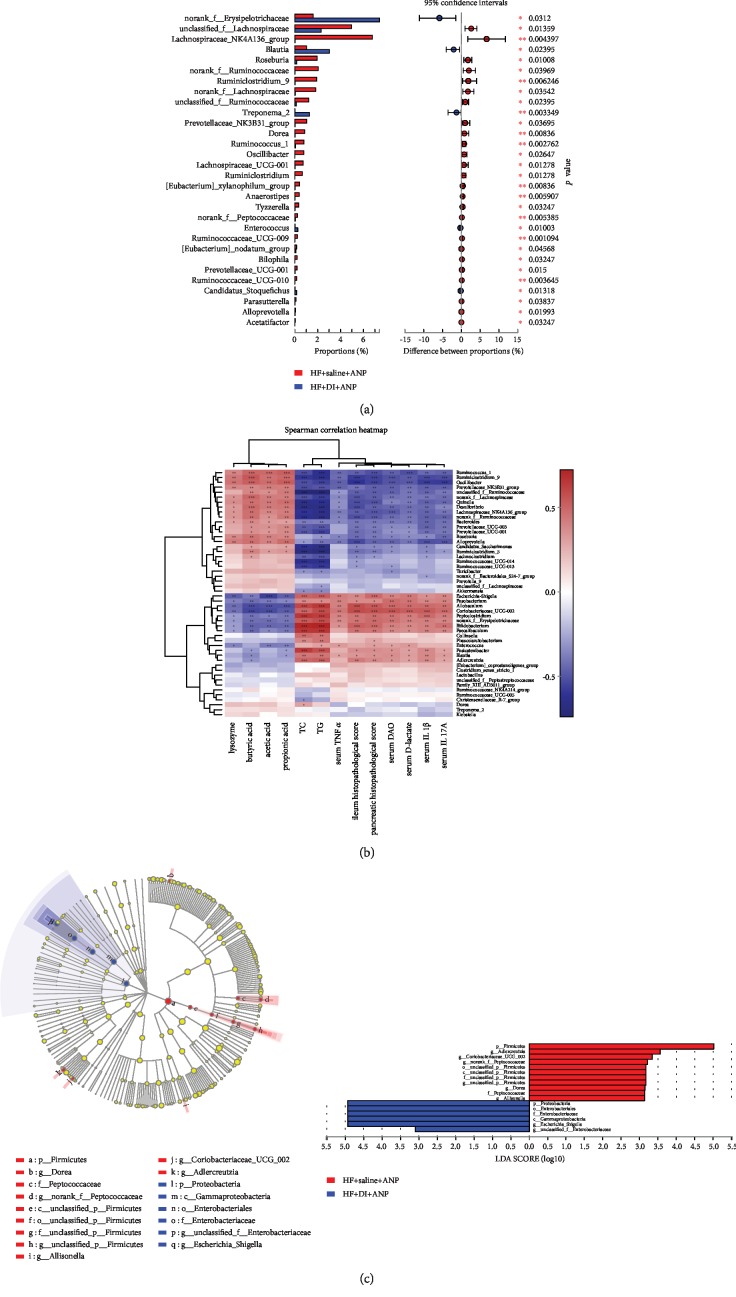
Effects of dithizone on microbiota composition in rats on high-fat diet followed by retrograde sodium taurocholate infusion. (a) Bacterial phyla and genera with significantly different abundance in rats treated with dithizone or saline. ^∗^*p* < 0.05, ^∗∗^*p* < 0.01, ^∗∗∗^*p* < 0.001, Wilcoxon test. (b) Heatmap showing correlation among SCFAs, lysozyme, intestinal barrier dysfunction, serum lipid, pathological changes, and gut microflora. Blue indicates a negative correlation, while red indicates a positive correlation. ^∗^*p* < 0.05, ^∗∗^*p* < 0.01, ^∗∗∗^*p* < 0.001. Spearman test. (c) LEfSe multilevel species hierarchy tree and LEfSe bars discriminated by LDA. *n* = 8 for each group.

Figures 8(c) and 8(d) show the fecal microbial composition in DI+HF and HF rats. Compared to the HF group, rats in the DI+HF group had enrichment of *Firmicutes* (from phylum to genus), *Peptococcaceae* (family), *Allisonella* (genus), *Adlercreutzia* (genus), and *Dorea* (genus) (Figure 9(c)). There was also enrichment of *Proteobacteria* (from phylum to family) and *Escherichia_shigella* (genus) in HF rats compared to DI+HF rats. At the phylum level, DI+HF rats had more *Spirochaetae* than HF rats (Figure 9(a)). The following genera had lower enrichment in DI+HF rats than HF rats: *Dorea*, *Anaerostipes*, *Tyzzerella*, *Oscillibacter*, *Bilophila*, *Parasutterella*, *Acetatifactor*, *Lachnospriraceae*_NK4A136_group, *Prevotellaceae*_NK3B31_group, *Ruminiclostridium*_9, *Ruminococcus*_1, *Roseburia*, and *Alloprevotella* (Figure 9(a)). In contrast, *Blautia*, *Candidatus_stoquefichus*, *Treponena*_2, and *Enterococcus* were higher in DI+HF rats than HF rats (Figure 9(a)). DI+HF rats also had lower levels of fecal SCFAs than HF rats at 6 and 24 h (Figure 3(b)).

When ANP was not induced (sham induction), DI+HF rats showed a trend towards lower intestinal microbial diversity than HF rats, but this was not statistically significant (Supplementary [Supplementary-material supplementary-material-1]). PCA analysis showed that DI+HF and HF rats had different microbial compositions even without ANP induction (Supplementary [Supplementary-material supplementary-material-1]). However, there was no significant difference in fecal SCFAs at 6 and 24 h except for n-valeric acid (Supplementary [Supplementary-material supplementary-material-1]).

Spearman's rank correlation showed that *Desulfovibrio*, *Alloprevotella*, *Oscillibacter*, *Quinella*, *Bacteroides*, *Lachnospiraceae*_NK4A136_group, *Prevotellaceae*_NK3B31_group, *Ruminococcus*_1, and *Ruminiclostridium*_9 all positively correlated with SCFAs and lysozyme expression, while they negatively correlated with TC, TG, DAO activity, serum D-lactate, serum inflammatory cytokines, and pathological injuries (Figure 9(b)). In contrast, *Allobaculum*, *Bifidobacterium*, *Escherichia-Shigella*, *Fusobacterium*, and *Peptoclostridium* negatively correlated with SCFAs and lysozyme expression, while they positively correlated with TC, TG, DAO activity, serum D-lactate, serum inflammatory cytokines, and pathological injuries (Figure 9(b)).

## 4. Discussion

In this study, we show that depleting Paneth cells using dithizone exacerbates pancreatic pathology, inflammation, and intestinal injury in rat models of ANP after normal and high-fat diet.

High-fat feeding exacerbated pancreatic and intestinal ANP-associated injuries. This is consistent with previous studies showing that hypertriglyceridemia is a crucial factor that exacerbates pancreatic pathology and intestinal injuries in ANP [5, 16, 26]. Several studies have demonstrated that over 8-week high-fat diet decreased tight-junction proteins and increased intestinal permeability in mice [14, 27, 28]. In our current study, we found 2-week high-fat diet decreased TJ proteins and increased intestinal permeability in ANP rats, which was consistent with our previous study [5]. However, the mechanism is not clear. High-fat feeding also reduced fecal microbial diversity, in agreement with the findings of previous studies [11, 29]. These observations are likely linked to the decrease in Paneth cell number and lysozyme expression in response to high-fat feeding. Lysozyme is an essential effector secreted by Paneth cells and its expression is known to be correlated with microbiota homeostasis [30].

Paneth cell ablation with dithizone aggravated pancreatic and intestinal injuries in rats with ANP on standard or high-fat diets. Paneth cell depletion in mice has been reported to cause intestinal barrier damage and enhanced pathogen transmission [31]. Translocation of bacteria from the small bowel [32] can aggravate damage to the intestinal barrier in ANP [33], and intestinal barrier dysfunction is a major contributor to ANP complications [34]. In contrast, Paneth cell depletion alone (without ANP induction) had no significant effect on pancreatic and intestinal injuries or intestinal microbiota composition. This is consistent with previous studies showing that depletion of Paneth cells with dithizone does not affect the intestinal barrier [35].

Paneth cell depletion also changed intestinal microbiota structure and decreased microbial diversity in rats with ANP after normal and high-fat diet. Paneth cells directly sense commensal microbiota through a TLR-MyD88-dependent pathway [36] and regulate innate immunity in the intestine [7]. Intestinal microbiota is an irreplaceable factor in maintaining intestinal homeostasis [37]. Probiotics can prevent DSS-induced intestinal injuries by strengthening intestinal barrier integrity [38]. A recent study shows that interactions between diet and intestinal microbiota can modify intestinal permeability and colitis severity in mice [39].

Analysis of the microbial phylogenetic composition showed that *Bacteroidetes* was decreased and *Actinobacteria* was increased in rats on standard diet, while *Spirochaetae* was increased in rats on high-fat diet. *Bacteroidetes* is associated with high-fat feeding and chronic respiratory inflammation [40, 41]. Several bacterial genera including *Alloprevotella*, *Oscillibacter*, *Quinella*, *Ruminococcus*_1, *Ruminiclostridium*_9, and *Escherichia-Shigella* also correlated with increased intestinal permeability, inflammation, and pancreatic and ileal injury in rats fed standard or high-fat diets. *Alloprevotella* has been reported to negatively correlate with inflammation and positively correlate with HDL-C in rats with type 2 diabetes [42], while *Allobaculum* negatively correlates with serum alanine aminotransferase in mice with nonalcoholic fatty liver disease [43].

Paneth cell depletion decreased fecal SCFAs in ANP rats on standard or high-fat diets. Several bacterial genera were also correlated with SCFAs, in agreement with the findings of a previous study [42]. SCFAs are known to regulate intestinal barrier integrity and inflammatory responses through GPR41 and GPR43 [44]. Previous studies have reported that SCFAs act as histone deacetylase inhibitors to suppress the inflammatory response, NLRP3 inflammasome activity and autophagy [45]. We speculate that several bacterial genera may exert anti-inflammatory effects by regulating SCFAs.

Paneth cell ablation aggravates pancreatic and intestinal injuries after normal and high-fat diet. Meanwhile, intestinal microbiota and its metabolites are greatly changed. As shown in Supplementary [Supplementary-material supplementary-material-1], we hypothesized that the mechanism by which Paneth cell ablation aggravates ANP after normal or high-fat diet may be related to intestinal barrier dysfunction, proinflammatory cytokines, microbiota dysbiosis, and short-chain fatty acids. However, its mechanism is still unclear. We will explore the role of Paneth cells in ANP in the further studies.

## 5. Conclusions

Paneth cell depletion enhances pancreatic and intestinal injuries in a rat model of ANP on standard or high-fat diets. Changes in microbiota and SCFAs are associated with pancreatic and intestinal injury. Our study sheds further light on the associations among Paneth cells, intestinal microbiota, and ANP. SCFAs produced by microbiota may have potential as new therapeutic targets for treating ANP.

## Data Availability

The data used to support the findings of this study are available from the corresponding authors upon request. The sequence data generated in this study were submitted to the GenBank Sequence Read Archive accession number PRJNA540021.

## Supplementary Materials

Supplementary Materials Supplementary Table 1: pancreatic lesion index (scoring criteria). Supplementary Table 2: ileal lesion index (scoring criteria). Supplementary Figure 1: dithizone depletes Paneth cells in rats. Supplementary Figure 2: effect of high-fat diet on ANP-associated injuries caused by retrograde sodium taurocholate infusion. Supplementary Figure 3: effects of dithizone in rats on standard diet followed by sham biliopancreatic infusion. Supplementary Figure 4: effects of dithizone in rats on high-fat diet followed by sham biliopancreatic infusion. Supplementary Figure 5: mechanism of Paneth cells in pancreatic and intestinal injuries. Supplementary Figure 6: images of the control reaction (tissue sections where the primary antibody was omitted during the immunohistochemistry reaction).
