# Supplementary material for: Paneth Cell Ablation Aggravates Pancreatic and Intestinal Injuries in a Rat Model of Acute Necrotizing Pancreatitis after Normal and High-Fat Diet
Source: Mediators Inflamm. 2019 Nov 11;2019:8474523. doi: 10.1155/2019/8474523 (PMC6885277; doi:10.1155/2019/8474523)
Supplement: Supplementary Materials — Supplementary Table 1: pancreatic lesion index (scoring criteria). Supplementary Table 2: ileal lesion index (scoring criteria). Supplementary Figure 1: dithizone depletes Paneth cells in rats. Supplementary Figure 2: effect of high-fat diet on ANP-associated injuries caused by retrograde sodium taurocholate infusion. Supplementary Figure 3: effects of dithizone in rats on standard diet followed by sham biliopancreatic infusion. Supplementary Figure 4: effects of dithizone in rats on high-fat diet followed by sham biliopancreatic infusion. Supplementary Figure 5: mechanism of Paneth cells in pancreatic and intestinal injuries. Supplementary Figure 6: images of the control reaction (tissue sections where the primary antibody was omitted during the immunohistochemistry reaction). [file 8474523.f1.pdf]

Pancreatic histologic scoring criteria (3 slides/animal)				
Scores	Edema	Inflammation and Infiltration	Acinar Necrosis	Hemorrhage
0	Absent	0-1 Intralobular or perivascular leukocytes/HPF	Absent	Absent
0.5	Focal expansion of interlobar septae	2-5 Intralobular or perivascular leukocytes/HPF	Focal occurrence of 1-4 necrotic cells/HPF	1 focus
1	Diffuse expansion of interlobar septae	6-10 Intralobular or perivascular leukocytes/HPF	Diffuse occurrence of 1-4 necrotic cells/HPF	2 foci
1.5	Same as 1+ focal expansion of interlobar septae	11-15 Intralobular or perivascular leukocytes/HPF	Same as 1+ focal occurrence of 5-10 necrotic cells/HPF	3 foci
2	Same as 1+ diffuse expansion of interlobar septae	16-20 Intralobular or perivascular leukocytes/HPF	Diffuse occurrence of 5-10 necrotic cells/HPF	4 foci
2.5	Same as 2+ focal expansion of interacinar septae	21-25 Intralobular or perivascular leukocytes/HPF	Same as 2+ focal occurrence of 11-16 necrotic cells/HPF	5 foci
3	Same as 2+ diffuse expansion of interacinar septae	26-30 Intralobular or perivascular leukocytes/HPF	Diffuse occurrence of 11-16 necrotic cells/HPF	6 foci
3.5	Same as 3+ focal expansion of intercellular spaces	>30 leukocytes/HPF or focal microabscesses	Same as 3+ focal occurrence of >16 necrotic cells/HPF	7 foci
4	Same as 3+ diffuse expansion of intercellular spaces	>35 leukocytes/HPF or confluent microabscesses	>16 necrotic cells/HPF	8+ foci

Supplementary Table 1: Pancreatic lesion index (scoring criteria)

Ileal histologic scoring criteria (3 slides/animal)			
Scores	Mucosal damage	Inflammation	Congestion/Hemorrhage
0	Absent	Absent	Absent
1	Increase of spaces under intestinal mucosal epithelium, with capillary congestion and villi swelling	Focal occurrence of inflammatory cells in the lamina propria	Capillary congestion in the lamina propria
2	Expansion of spaces under intestinal mucosal epithelium, with moderate separation between epithelial layer and lamina propria	Diffuse occurrence of inflammatory cells in the lamina propria	Focal hemorrhage in the lamina propria
3	Extensive expansion of spaces under intestinal mucosal epithelium, with broken villi top	Focal occurrence of inflammatory cells under the endothelium	Diffuse hemorrhage in the lamina propria
4	Villus breakage and shedding of lamina propria, with exposure of lamina propria capillaries	Diffuse occurrence of inflammatory cells under the endothelium	Subendothelial hemorrhage
5	Degeneration of the lamina propria, accompanied by bleeding and ulceration	Extensive accumulation of Inflammatory cells	Extensive hemorrhage

Supplementary Table 2: Ileal lesion index (scoring criteria)

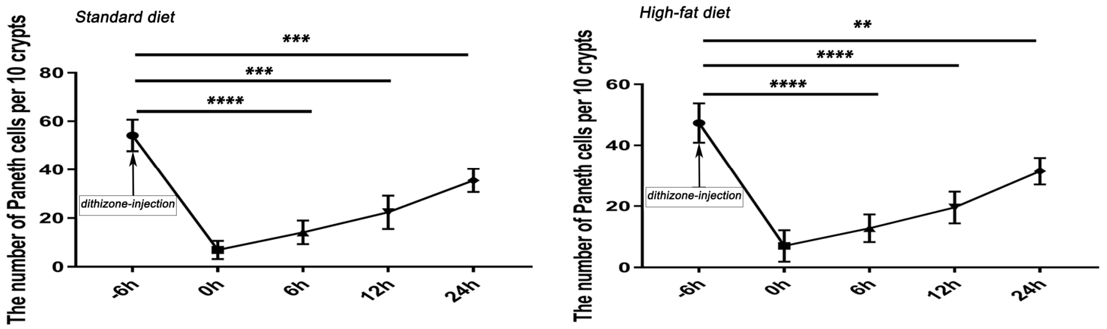

Supplementary Figure 1: Dithizone depletes Paneth cells in rats. Quantification of Paneth cells after dithizone injection in rats on a standard or high-fat diet. *p<0.05, **p<0.01, ***p<0.001, ****p<0.0001, one-way ANOVA test. Results were expressed as the mean ± SD (n=8).

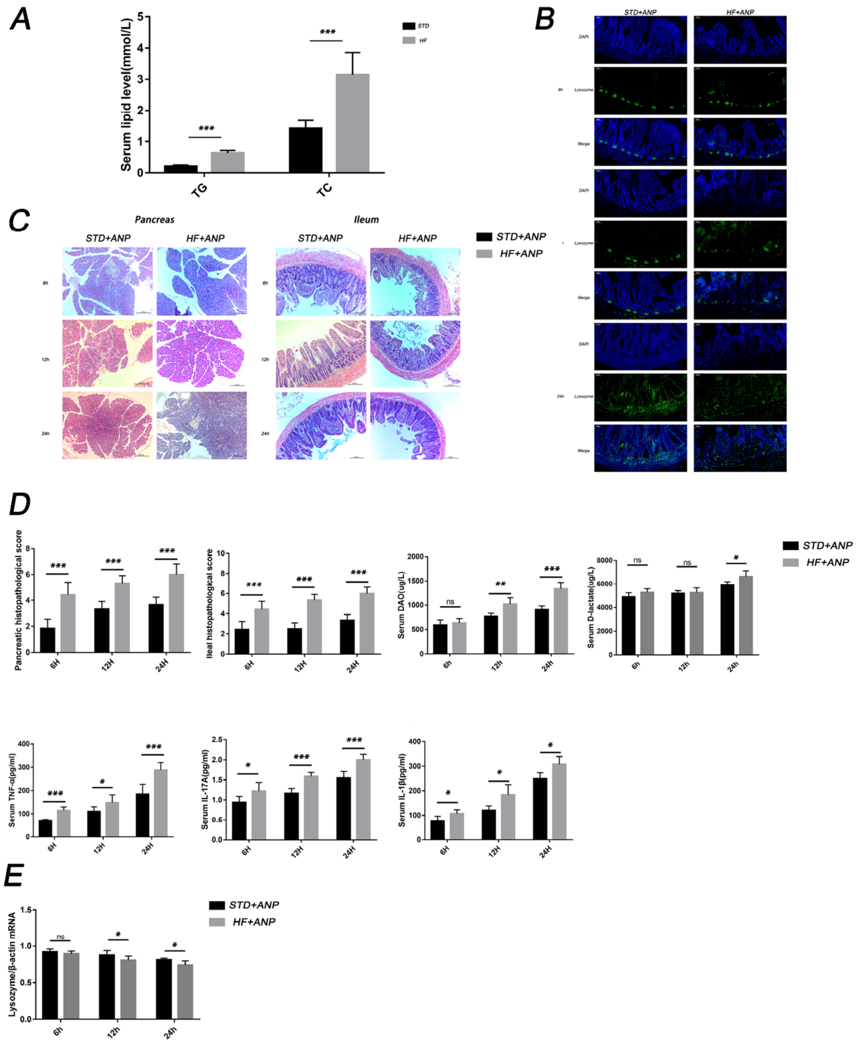

Supplementary Figure 2: Effect of high-fat diet on ANP-associated injuries caused by retrograde sodium taurocholate infusion. (A) Serum TG and TC levels in rats fed a high-fat or standard diet. (B) Lysozyme protein expression (green) in Paneth cells of the distal ileum as assessed by immunofluorescence. Nuclei were counterstained with DAPI (magnification ×200). (C) Pancreas and distal ileum sections of rats fed high-fat or standard diet stained with H&E (magnification ×100). (D) Histopathological scores of pancreas and distal ileum, serum levels of inflammatory cytokines, diamine oxidase (DAO) activity and serum D-lactate in rats fed a high-fat or standard diet. (E) Lysozyme mRNA expression as assessed by RT-PCR. *$p<0.05$, **$p<0.01$, ***$p<0.001$, Student's t-test. Results were expressed as the mean ± SD (n=5).

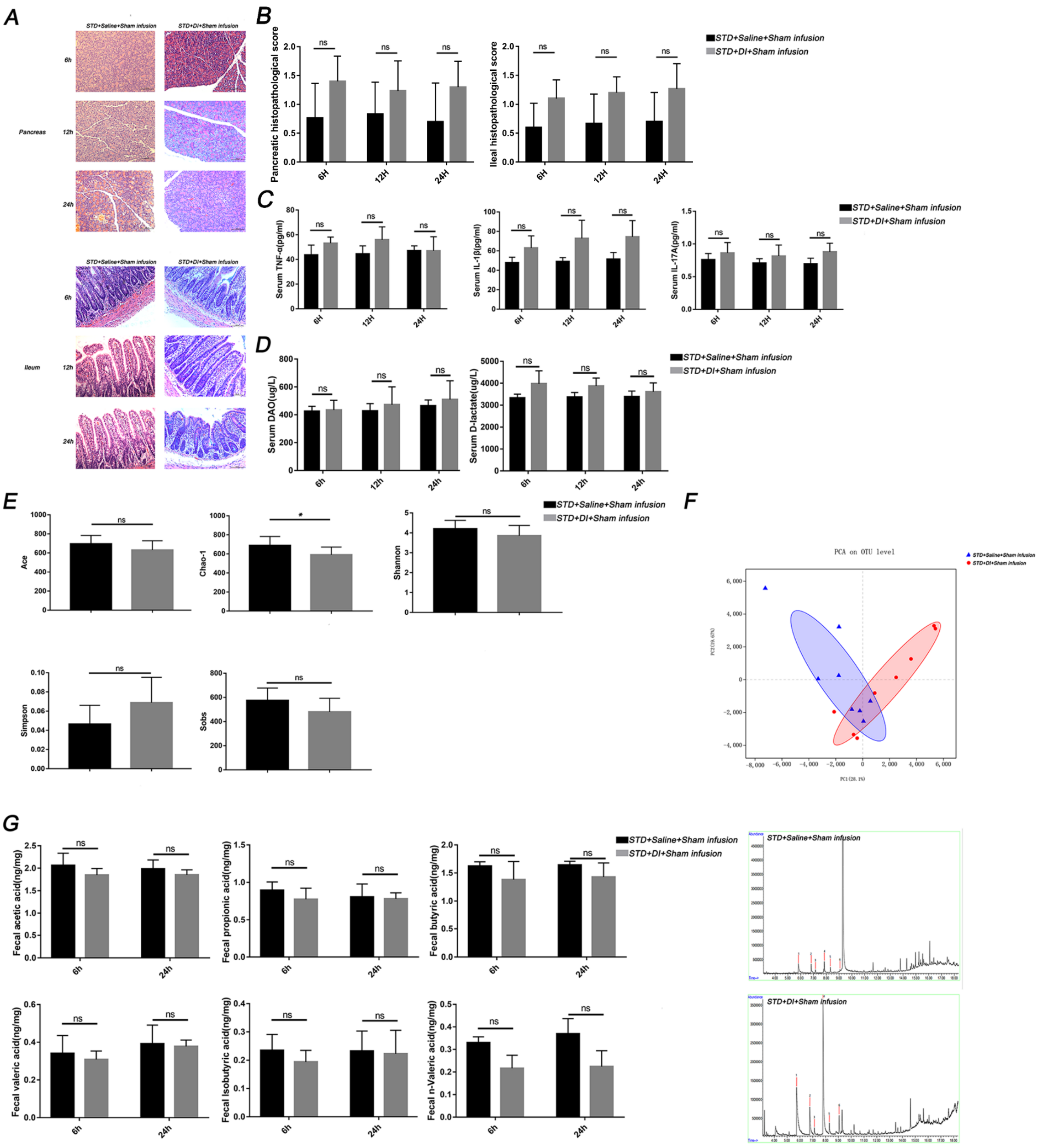

Supplementary Figure 3: Effects of dithizone in rats on standard diet followed by sham biliopancreatic infusion. (A) Sections of pancreas and the distal ileum stained with H&E in rats treated with dithizone or saline (magnification ×200). (B) Histopathological scores of the pancreas and the distal ileum. (C) Serum inflammatory cytokines in rats treated with dithizone or saline. (D) DAO activity and serum D-lactate in rats treated with dithizone or saline. (E) Estimators of intestinal

microbiota α-diversity in rats treated with dithizone or saline. (F) PCA of microbiota β-diversity in rats treated with dithizone or saline. (G) Fecal SCFA levels in rats treated with dithizone or saline. *$p<0.05$, Student's t-test. Results were expressed as the mean ± SD (n=5).

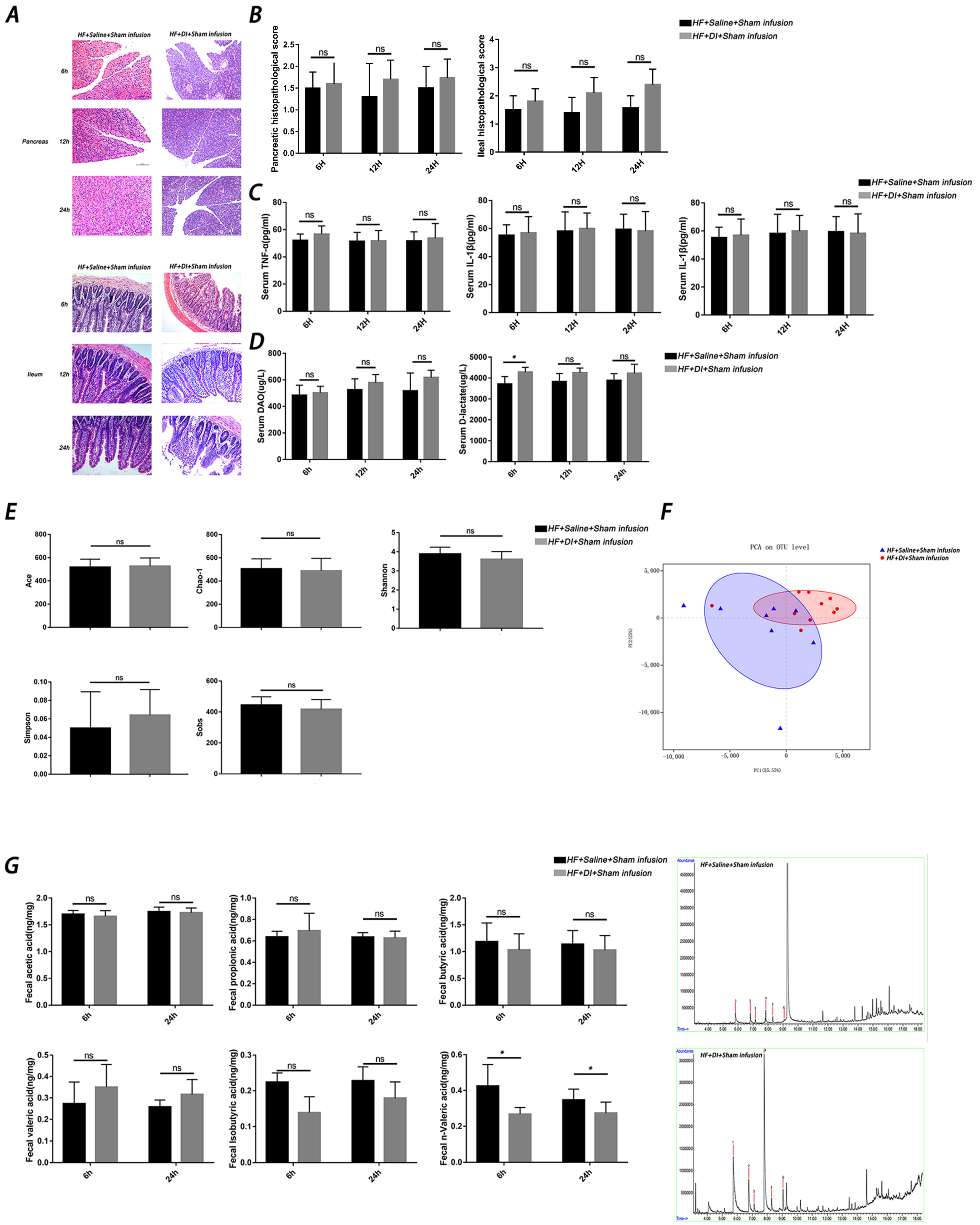

Supplementary Figure 4: Effects of dithizone in rats on high-fat diet followed by sham biliopancreatic infusion. (A) Sections of pancreas and distal ileum stained with H&E in rats treated with dithizone or saline (magnification ×200). (B) Histopathological scores of the pancreas and the distal ileum. (C) Serum inflammatory cytokines in rats treated with dithizone or saline. (D) DAO activity and serum D-lactate in rats treated with dithizone or saline. (E) Estimators of intestinal microbiota α-diversity in rats treated with dithizone or saline. (F) PCA of microbial β-diversity in rats treated with dithizone or saline. (G) Fecal SCFA levels in rats treated with dithizone or saline. **p*<0.05, Student's t-test. Results were expressed as the mean ± SD (n=5).

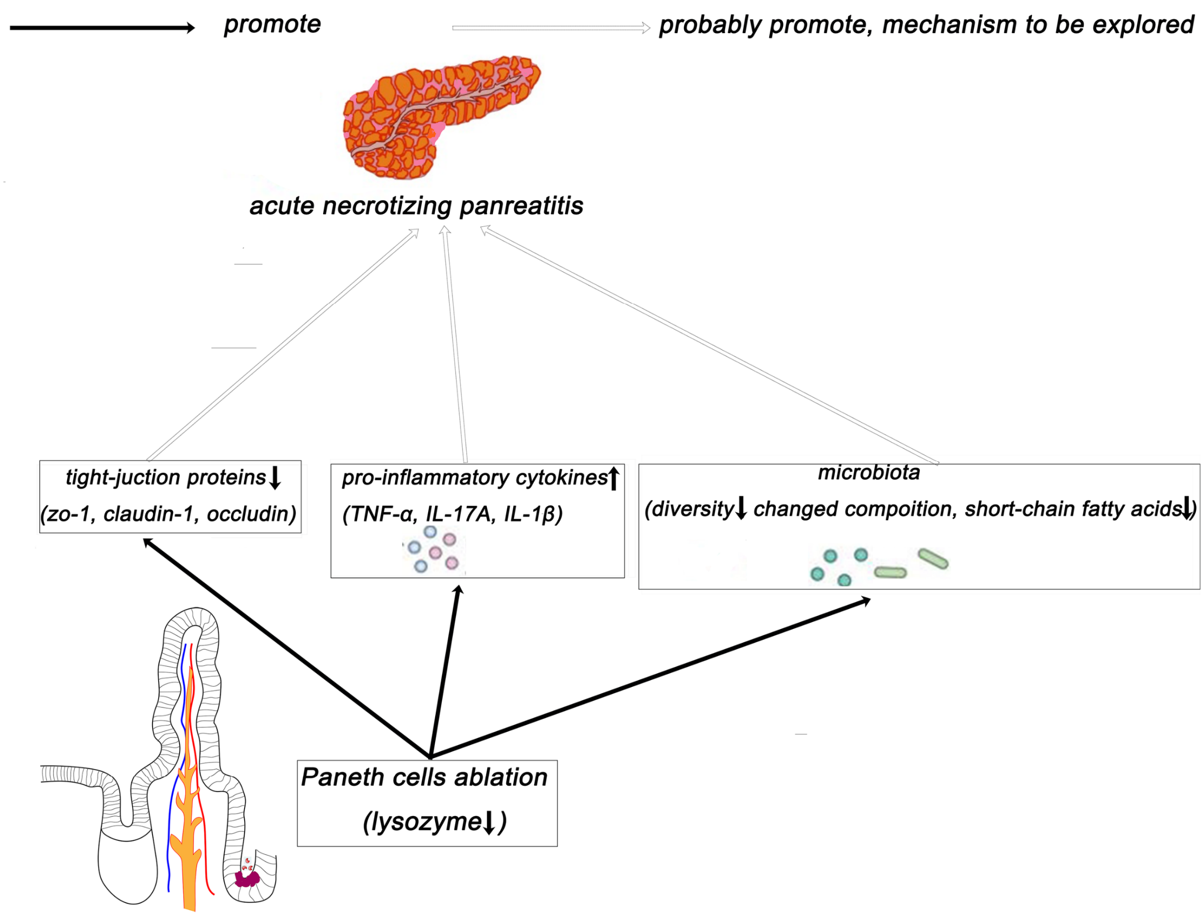

Supplementary Figure 5: Mechanism of Paneth cells in pancreatic and intestinal injuries.

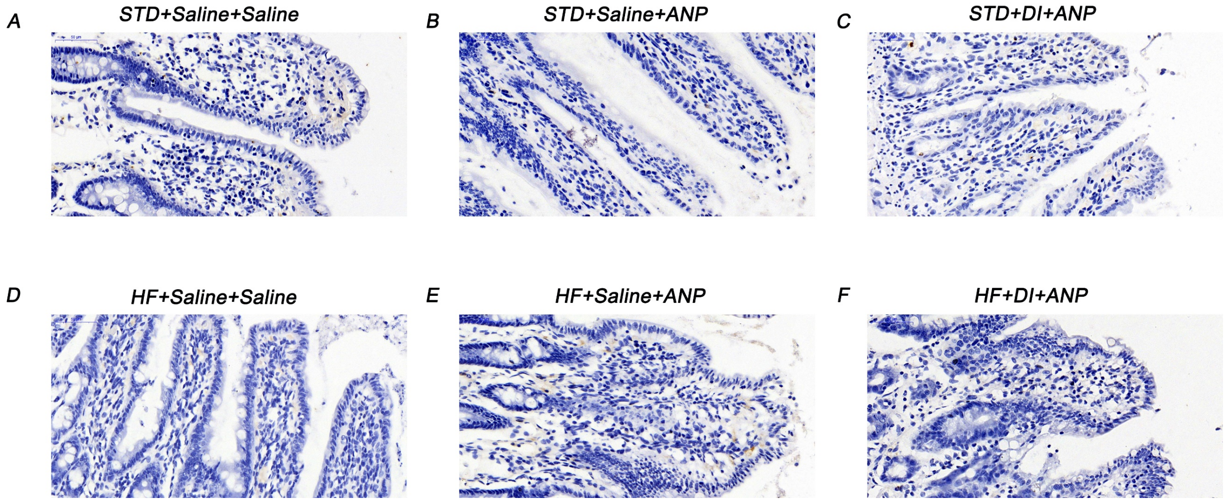

Supplementary Figure 6: Images of the control reaction (tissue sections where the primary antibody was omitted during the immunohistochemistry reaction). (A) Group STD + Saline + Saline. (B) Group STD + Saline + ANP. (C) Group STD + DI + ANP. (D) Group HF + Saline + Saline. (E) Group HF + Saline + ANP. (F) Group HF + DI + ANP.
